# Selection signatures associated with adaptation in South African Drakensberger, Nguni, and Tuli beef breeds

**DOI:** 10.1007/s11250-024-04265-8

**Published:** 2024-12-27

**Authors:** Gomo Maxman, Este van Marle-Köster, Simon Frederick Lashmar, Carina Visser

**Affiliations:** 1 Department of Animal Science, Faculty of Natural & Agricultural Sciences, University of Pretoria, Pretoria, South Africa; 2 Animal Production, Agricultural Research Council, Pretoria, South Africa

**Keywords:** Adaptation, Candidate genes, Indigenous breeds, Runs of homozygosity, Selection signatures

## Abstract

In the present study 1,709 cattle, including 1,118 Drakensberger (DRB), 377 Nguni (NGI), and 214 Tuli (TUL), were genotyped using the GeneSeek® Genomic Profiler™ 150 K bovine SNP panel. A genomic data set of 122,632 quality-filtered single nucleotide polymorphisms (SNPs) were used to identify selection signatures within breeds based on conserved runs of homozygosity (ROH) and heterozygosity (ROHet) estimated with the *detectRUNS* R package. The mean number of ROH per animal varied across breeds ranging from 36.09 ± 12.82 (NGI) to 51.82 ± 21.01 (DRB), and the mean ROH length per breed ranged between 2.31 Mb (NGI) and 3.90 Mb (DRB). The smallest length categories i.e., ROH < 4 Mb were most frequent, indicating historic inbreeding effects for all breeds. The ROH based inbreeding coefficients (F_ROH_) ranged between 0.033 ± 0.024 (NGI) and 0.081 ± 0.046 (DRB). Genes mapped to candidate regions were associated with immunity (*ADAMTS12, LY96, WDPCP*) and adaptation (*FKBP4, CBFA2T3, TUBB3*) in cattle and genes previously only reported for immunity in mice and human (*EXOC3L1*, *MYO1G*). The present study contributes to the understanding of the genetic mechanisms of adaptation, providing information for potential molecular application in genetic evaluation and selection programs.

**Supplementary Information:**

The online version contains supplementary material available at 10.1007/s11250-024-04265-8.

## Introduction

In Africa, livestock is integral to food security, as well as social, cultural, and economic development (Mapiye et al. 2019). However, the looming threat of climate change has the potential to adversely affect future food production (Godde et al. 2021). Climate change effects on livestock farming include water scarcity, altered precipitation patterns, and extreme weather events that have the potential to disrupt agricultural production (Gomez-Zavaglia et al. 2020). The demand for animal protein is escalating, particularly in developing countries of sub-Saharan Africa that will, according to projections, contribute to more than 50% of the global population growth by 2050 (United Nations 2022).

The South African (SA) beef sector comprises a variety of cattle breeds classified as either exotic (indicine, and taurine), indigenous (Sanga), or composite (Strydom 2008). Indigenous Sanga cattle are a combination of African taurine, African indicine and European taurine (Makina et al. 2016). In SA, approximately 53% of beef cattle are raised in commercial systems, with the remaining 47% being reared under informal conditions (Department of Agriculture, Land Reform and Rural Development 2021). Sanga cattle play a significant role in both the well-developed (commercial) and developing (non-commercial, and informal) livestock production systems across the country (Van Marle-Köster and Visser 2018) and may harbour unique traits due to both artificial and natural selection. They are known for their unique adaptive traits that include tolerance to endemic diseases, internal and external parasites (Mapholi 2015; Mapholi et al. 2022), extreme temperatures (Nyamushamba et al. 2017), changes in feed availability, and low management inputs (Scholtz 2010). According to the Department of Agriculture, Land Reform and Rural Development (2021), 29% of the total meat produced under intensive feedlot conditions originated from Sanga cattle types, signifying their contribution to the country’s beef production.

Initiated in 2015, the Beef Genomics Program (BGP) contributed to the establishment of reference populations for SA Sanga (Afrikaner, Drakensberger, Nguni, and Tuli,) and locally developed composite beef breeds (e.g., the SA Bonsmara). This program provided an influx of genomic data for these breeds, which allowed a multitude of genomic applications not possible before (SA Stud Book 2022). The availability of higher density data for larger numbers of cattle per breed (that are also more representative of the national herd), allows for a more precise detection of selection signatures through better capturing of changes in linkage disequilibrium (LD) and allele frequencies (Saravanan et al. 2021). Long-term selection (natural and/or artificial) changes specific regions of the genome resulting in selection signatures that can assist in identifying genes associated with certain traits (Moravčíková et al*.* 2018).

Runs of homozygosity (ROH) refer to stretches of homozygous genotypes that are frequently observed in individuals and populations who share a common ancestor (Gibson et al. 2006). Longer ROH can suggest recent inbreeding or a small effective population size (Marras et al. 2015; Doekes et al. 2019), while shorter ROH may indicate ancient inbreeding or a genetically diverse population (Forutan et al. 2018). Shared or consensus ROH within a population can be used to identify conserved genomic regions potentially under selection, which may be involved in defining breed-specific traits or the adaptation to the environment (Purfield et al. 2017). Several studies have used ROH to detect within-breed selection signatures in cattle (Mastrangelo et al. 2016; Yurchenko et al. 2018; Biscarini et al. 2020; Singh et al. 2020). In contrast, heterozygosity-rich regions (or runs of heterozygosity; ROHet) refers to stretches of the genome where an individual inherits different alleles from each parent and may serve as an indicator of genetic diversity within the population (Kenny et al. 2022). This concept is new and has received limited attention in animal-based genomics studies, with only a few studies conducted on cattle (e.g., Biscarini et al. 2020; Lashmar et al. 2022). Despite benchmark studies on the distribution and characteristics of ROH, ROHet and selection signatures within and across SA indigenous cattle (Makina et al. 2016; Lashmar et al. 2018; King et al. 2022; Kooverjee et al. 2022), the genetic architecture of adaptive traits that are unique to these populations remains only partially understood.

The aim of this study was to assess the distribution and characteristics of ROH and ROHet and to identify selection signatures associated with adaptation within and between the Drakensberger (DRB), Nguni (NGI), and Tuli (TUL) populations.

## Materials and methods

Single nucleotide polymorphism (SNP) genotypes were available for 1,722 individuals representing the SA Tuli (216), Nguni (381) and Drakensberger (1,125) breeds**.** These animals were genotyped with the GeneSeek® Genomic Profiler™ 150 K bovine SNP panel (140,113 SNPs) (Illumina Inc. San Diego, CA, USA: www.illumina.com) as part of the BGP at the Agricultural Research Council’s Biotechnology Platform (ARC-BTP). Genotype quality control (QC) procedures were performed using PLINK version 1.9 software package (Purcell et al. 2007). Only unique (i.e. non-duplicated), autosomal SNP markers were considered for further analysis. For ROH analyses, individuals with a sample call rate below 90%, markers with a SNP call rate below 95%, and deviations from Hardy–Weinberg equilibrium (*P* < 0.001) were excluded from the analyses. No MAF pruning was performed to avoid the removal of SNPs that are either fixed or highly homozygous (Meyermans et al. 2020). The same QC measures were applied for ROHet and F_ST_ analyses, except that MAF pruning (MAF < 5%) was performed to prevent underestimation of the length of segments (Meyermans et al. 2020). No LD pruning was performed for any analyses. Following these QC steps, 1,709 animals (DRB: 1,118; NGI: 377; TUL: 214) and 122,632 common SNPs remained for downstream analyses.

The consecutive-SNP-based detection method of the *detectRUNS* R-package version 0.96 (Biscarini et al. 2018; Saravanan et al. 2021), was used to identify ROH and ROHet. To minimize the risk of spurious ROH due to low SNP densities, the following criteria were applied: a minimal ROH length set to 1 Mb; a maximum distance between SNPs equal to 1 Mb; no SNPs with a heterozygous genotype were allowed, and a maximum of 2 SNPs with a missing genotypes were allowed (Biscarini et al. 2020). The minimum number of SNPs constituting a ROH segment (l = 52 DRB; l = 54 NGI; l = 50 TUL) was estimated using Purfield et al. (2012):

$$l=\frac{{log}_{e} \frac{\alpha }{{n}_{s}{n}_{i}}}{{log}_{e} \left(1-\overline{het }\right)},$$

where n_s_ and n_i_ were the numbers of SNPs and individuals, respectively, α (set to 0.05) represented the proportion of false positive identifications, and het was the mean SNP-wide heterozygosity.

The mean number and length of ROH were calculated per animal and the ROH were grouped into four classes: ROH < 4 Mb, 4 ≤ ROH < 8 Mb, 8 ≤ ROH < 16 Mb, and greater than 16 Mb. The genomic inbreeding coefficient (F_ROH_) was calculated based on McQuillan et al*.* (2008):

$${\text{F}}_\text{ROH}=\frac{S_{ROH}}{L_{GEN}};$$

where ${{\varvec{L}}}_{{\varvec{G}}{\varvec{E}}{\varvec{N}}}$ and ${{\varvec{S}}}_{{\varvec{R}}{\varvec{O}}{\varvec{H}}}$ represented the base pair length of the genome covered by SNPs and the summed length of ROH per animal, respectively.

The total sum of ROH lengths in the selected ROH length category for each individual was divided by the sum of lengths of autosomal chromosomes covered by SNPs. The average F_ROH_ was estimated for various ROH length categories: ROH < 4 Mb (F_ROH<4 Mb_), 4 Mb ≤ ROH < 8 Mb (F_ROH=4-8 Mb_), 8 Mb ≤ ROH < 16 Mb (F_ROH=8-16 Mb_) and ROH ≥ 16 Mb (F_ROH≥16 Mb_).

Given the absence of a consensus standard criteria for ROHet analysis and the scarcity of published studies, the criteria proposed by Biscarini et al. (2020), (Santos et al. 2023) and Li et al*.* (2022) were applied. These criteria include: the default minimum of 15 SNPs constituting an ROHet; a minimal ROHet length set to 250 Kb; a maximum distance between SNPs equal to 1 Mb; a maximum of 3 SNPs with homozygous genotypes; and a maximum of 2 SNPs with missing genotypes. The ROHet were grouped into the following length categories: ROHet < 0.25 Mb, 0.25 ≤ ROHet < 0.5, 0.5 ≤ ROHet < 1, 1 ≤ ROHet ≤ 2, and ROHet > 2 Mb (Biscarini et al. 2020). The number, proportion, and mean length of ROHet were calculated within each length category per breed.

To identify within-breed selection signatures, the *detectRUNS* R-package version 0.96 (Biscarini et al. 2018) was used to identify genomic regions most commonly associated with ROH and ROHet (ROH and ROHet islands). The percentage occurrence of a SNP in ROH and ROHet was calculated by counting the number of times the SNP appeared in those ROH and ROHet across animals within each population. To identify ROH and ROHet islands, SNPs falling within 99th percentile of the locus homozygosity and heterozygosity distribution were selected. This translated to an in-ROH and in-ROHet frequency threshold of 0.2 and 0.3, respectively (Biscarini et al. 2020). The function “topRuns” from the *detectRUNS* package was used to retrieve the most common runs using these threshold values. This means that a ROH and ROHet had to be present in at least 20% and 30% of each population to be included in an ROH or ROHet island, respectively.

The F_ST_ was used to estimate the genetic differentiation between populations using the formula proposed by Nei (1986):

$${F}_{ST}=\frac{{H}_{T}-{H}_{S}}{{H}_{T}}$$

Where,

F_ST_: the reduction in heterozygosity due to the structure of the population
H_S_: Average heterozygosity in the subpopulation
H_T_: Average heterozygosity in the metapopulation

The three beef cattle populations were compared pairwise (i.e. DRB-versus-NGI, DRB-versus-TUL, and NGI-versus-TUL) using PLINK’s –fst command (Purcell et al. 2007) to determine genomic regions that exhibit differentiation. Negative F_ST_ values were set to 0 since they do not have any biological meaning (Akey et al. 2002). The top 0.1% F_ST_ values indicated selection signatures as suggested by Kijas et al. (2012), Zhao et al. (2015), and Saravanan et al. (2021). Prior to annotation, windows of 250 kb downstream and upstream of the significant SNPs were investigated to verify overlapping gene segments (Maiorano et al*.* 2018).

After identifying significant regions under selection, annotation was done using the National Centre for Biotechnology Information (NCBI) database based on ARS-UCD2.0 bovine genome assembly (https://www.ncbi.nlm.nih.gov/datasets/genome/GCF_002263795.3/). Functional annotations were then assigned to genes through ShinyGO 0.77 (Ge et al. 2020) and the PANTHER version 16 software (http://www.pantherdb.org) (Mi et al. 2021). Genes with known functions were enriched for further analysis of molecular functions, biological processes, and cellular components.

## Results

The total number of ROH identified varied among the three populations, ranging between 10,260 (TUL) and 57,885 (DRB) (Table 1). The DRB population had the highest mean number of ROH per animal (51.82 ± 21.01), as well as the longest ROH (71.9 Mb). The average genome-wide F_ROH_ values were highest in the DRB, followed by TUL, and NGI populations. The DRB had the most ROHet with 97,162, followed by NGI with 21,103, and the TUL with 16,368. A higher occurrence of ROHet < 0.25 Mb was observed compared to other size categories in all populations, and the number of ROHet decreased as the length of ROHet increased.

**Table 1:** Summary statistics of ROH and ROHet analyses in the three cattle populations

	Drakensberger	Nguni	Tuli
Mean nROH ± SD per individual	51.82 ± 21.01	36.09 ± 12.84	47.94 ± 15.36
Mean F_ROH±SD_	0.081 ± 0.046	0.033 ± 0.024	0.074 ± 0.031
Mean ROH length (Mb)	3.61	2.31	3.76
Longest ROH length (Mb)	71.9	65.5	53.8
ROH < 4 Mb	41,358	12,360	7,557
4 Mb ≤ ROH < 8 Mb	10,229	657	1,570
8 Mb ≤ ROH < 16 Mb	4,856	379	877
> 16 Mb	1,442	137	256
Total	**57,885**	**13,533**	**10,260**
Mean nROHet per individual	86.81 ± 11.73	55.98 ± 10.00	76.49 ± 12.33
Longest ROHet length (Mb)	2.84	1.60	1.48
Mean ROHet length (Mb)	0.3674	0.3673	0.3673
ROHet < 0.25 Mb	76,802	16,915	13,138
0.25 Mb ≤ ROHet < 0.5 Mb	19,655	4,053	3,132
0.5 Mb ≤ ROHet < 1 Mb	632	97	79
1 Mb ≤ ROHet < 2 Mb	72	38	19
ROHet > 2 Mb	1	-	-
Total	**97,162**	**21,103**	**16,368**

*SD* Standard deviation; *nROH* number of ROH; ***F***_***ROH***_ ROH-based Inbreeding coefficient, *nROHet* Number of Runs of heterozygosity

**Fig. 1:**
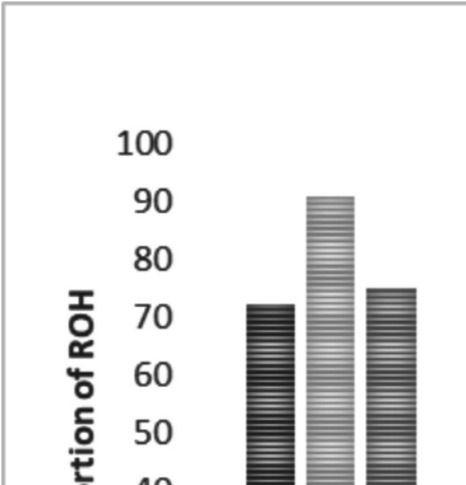
The proportion of ROH within various length categories in three cattle populations

The highest proportion of ROH across all populations, ranging from 71.45% (DRB) to 91.33% (NGI), was observed within the shortest length category (ROH < 4 Mb) (Fig. 1).

In the present study, three different approaches (ROH, ROHet and F_ST_) were used to detect candidate regions associated with adaptation and immunity. The distribution of ROH and ROHet islands across the chromosomes in three cattle breeds is depicted in Manhattan plots (Supplementary Figs. 1 A, B & C and 2 A, B, & C), respectively. The ROH islands harboured genes such as *LYZ, LYZ1, LYZ2,* and *LYZ3* related to immunity, and ROHet islands revealed candidate genes related to immunity (*ADAMTS12, CYSTM1, DTX1, WDPCP, ELMO3,* and *CBFA2T3*) and adaptation (*FKBP4* and *SRA1*). To visualize the genome-wide distribution of selection signatures for three breed pairs, the F_ST_ values for each SNP were plotted against their genomic position (Supplementary Fig. 3 A, B & C). Functional analyses of candidate regions within the top 0.1% of F_ST_ identified genes associated with immunity (*CDK10, LY96* and *CBFA2T3*) and adaptation traits (*LMAN2, TUBB3, NMU* and *MC1R*). Only genes associated with adaptation and immunity were discussed further (Table 2).

**Table 2:** List of candidate genes identified within genomic regions considered to be selection signatures using the ROH, ROHet, and F_ST_ approach in all three beef breeds

Gene ID	Population	Method	Ensembl Gene ID	BTA	Position (Mb)	Physiological Response
*MYO1G*	**TUL**	ROH	ENSBTAG00000006377	4	76.62	Immune response
*DDX56*		ROH	ENSBTAG00000010602	4	76.87	Immune response
*MYO1F*		ROH	ENSBTAG00000007661	7	17.12	Immune response
***LYZ1***	**TUL, DRBvsNGI**	*ROH, F*_*ST*_	ENSBTAG00000046511	5	44.39	Immune response
***LYZ2***		*ROH, F*_*ST*_	ENSBTAG00000026088	5	44.37	Immune response
***LYZ3***		*ROH, F*_*ST*_	ENSBTAG00000046628	5	44.42	Immune response
***LYZ***		*ROH, F*_*ST*_	ENSBTAG00000026779	5	44.51	Immune response
*FKBP4*	**DRB**	*ROHet*	ENSBTAG00000007605	5	106.95	Heat tolerance
*WDPCP*		*ROHet*	ENSBTAG00000005151	11	61.72	Immunity, High-altitude adaptation
*EXOC3L1*		*ROHet*	ENSBTAG00000012035	18	34.82	Immune response
*ELMO3*		*ROHet*	ENSBTAG00000001286	18	34.83	Immune response
*SLC4A9*	**TUL**	*ROHet*	ENSBTAG00000021775	7	51.55	Drought tolerance
***CYSTM1***	**NGI, TUL**	*ROHet*	ENSBTAG00000016596	7	51.36	Immune response
*SRA1*		*ROHet*	ENSBTAG00000001449	7	51.72	Immune response
*DTX1*		*ROHet*	ENSBTAG00000016738	17	61.09	Immune response
***ADAMTS12***		*ROHet*	ENSBTAG00000012558	20	39.87	Immune response
*NELL2*		*ROHet*	ENSBTAG00000032183	5	35.48	Immune response
***CBFA2T3***	**DRBvsNGI**	*F*_*ST*_	ENSBTAG00000010927	18	14.05	Immunity, High-altitude adaptation
*DEF8*		*F*_*ST*_	ENSBTAG00000039014	18	14.72	Immune response
***CDK10***	**DRBvsTUL**	*F*_*ST*_	ENSBTAG00000033333	18	14.57	Immune response
***MC1R***		*F*_*ST*_	ENSBTAG00000023731	18	14.71	Coat color
***TUBB3***		*F*_*ST*_	ENSBTAG00000023730	18	14.71	Coat color
*MTERF2*		*F*_*ST*_	ENSBTAG00000010144	18	70.25	Immune response
*LY96*		*F*_*ST*_	ENSBTAG00000008864	14	37.24	Immune response
*KCNE1*	**NGIvsTUL**	*F*_*ST*_	ENSBTAG00000001150	1	1.04	Immune response
*NMU*		*F*_*ST*_	ENSBTAG00000044161	6	71.03	Heat tolerance
*LMAN2*		*F*_*ST*_	ENSBTAG00000008034	7	38.83	Heat tolerance

***Bold*** = Genes observed in more than one population; *Mb* Megabase pairs; *DRB* Drakensberger; *NGI* Nguni; *TUL* Tuli

The study also identified shared candidate genes within genomic regions identified by all three approaches. The *FAM184B* gene, present in all three breeds, was identified through ROH analysis on BTA 6. The present study also identified a genomic segment on BTA 14 harbouring genes such as *LYN, SDCBP, CHD7*, and *CLVS1* common to all three approaches. These genes were not discussed further due to their prior links with feed efficiency and carcass traits.

## Discussion

In the present study, SNP genotypes were used to assess the distribution and characteristics of ROH and ROHet and identify selection signatures associated with adaptation within and between three SA indigenous cattle breeds. The distribution and number of ROH observed was consistent with expectations considering the genotypes available for each breed with the DRB (*n* = 1 118: *n*ROH = 57 885) exhibiting the most ROH, followed by NGI (*n* = 377: *n*ROH = 13 533) and TUL (*n* = 214: *n*ROH = 10 260). A high frequency of ROH < 4 Mb suggests that these runs originated from ancient generations (Peripolli et al. 2018) or recent admixture leading to the breakdown of longer ROH (Liu et al. 2021). However, Ferenčaković et al. (2013), indicated that medium density SNP arrays may overestimate shorter segments due to their limited sensitivity to detection. The high proportion of ROH > 16 Mb in the TUL breed suggests that recent inbreeding events may have occurred in this population, given that the TUL breed has the smallest population compared to other indigenous breeds in SA (SA Stud Book 2022). Despite the low proportions of ROH > 16 Mb, in NGI (0.101) and TUL (0.249) populations, routine monitoring is necessary especially in cases where high-impact bulls are used for mating.

The F_ROH_ is a reliable metric for assessing autozygosity, providing insights into both recent and ancient inbreeding patterns (Ferenčaković et al. 2013). In the ROH < 4 Mb category, higher F_ROH_ values, such as DRB (F_ROH<4 Mb_ = 0.055) and TUL (F_ROH<4 Mb_ = 0.049), suggest ancient inbreeding (Hulsegge et al. 2022). The F_ROH_ values align with the distribution of ROH in the DRB and TUL populations, showing decreasing F_ROH_ values with increasing ROH length.

Although ROHet regions remain unexplored compared to ROH (Tsartsianidou et al. 2021), they have been linked to loci that prevent the detrimental effects of continuous homozygosity and offer benefits for immunity, productivity, and reproduction (Chen et al. 2022; Ruan et al. 2022). ROHet are often concentrated in regions associated with disease resistance, where increased genetic diversity enhances populations’ ability to respond to health challenges, particularly novel threats (Sanglard et al. 2021). Similarly, in the present study, ROHet islands were associated with genes previously linked to heat tolerance (*FKBP4*), drought tolerance (*SLC4A9*), and immune response (e.g., *WDPCP, CYSTM1, SRA1,* and *ADAMTS12*). Genes such as *ADAMTS12* and *SRA1* have also been previously linked to other traits like body stature (Vanvanhossou et al. 2020) and fertility (Proto et al. 2024), respectively, highlighting the complexity of biological pathways and the need for further investigation.

The high prevalence of ROHet in the DRB is consistent with its known mixed genetic composition of European, African taurine, and indicine ancestry (Makina et al. 2016). Differences in ROHet prevalence between the DRB and other breeds (NGI and TUL) may be due to sample size variations. Unlike previous studies, e.g. (Biscarini et al. 2020; Lashmar et al. 2022), which reported higher ROHet proportions for the 0.5—1 Mb category, the current study found the highest mean proportion for ROHet ≤ 0.25 Mb across all populations, indicating selection for heterozygosity.

Katiyatiya et al. (2017) reported that the Nguni cows are more adapted to hot environments compared to Boran cows, maintaining a better thermal balance between body temperature and the environment. In the current study, three heat stress-related genes namely *FKBP4, LMAN2,* and *NMU* were identified in the three populations. The three genes are involved in thermoregulation, thermotolerance, and heat shock protein binding, interacting with proteins such as HSP27, HSP70, and HSP90 in cattle (Yayou et al. 2009; Archana et al. 2017; Saravanan et al. 2021). The *FKBP4* gene was upregulated in the heat shock pathway (GO:0031072), while the *LMAN2* gene was identified in enriched pathways of granulosa cells, influencing oocyte development in lactating cows exposed to various levels of hyperthermia (Klabnik et al. 2022). These genes highlight the genetic mechanism underlying heat tolerance and resilience in the studied populations.

The SA Drakensberger cattle typically have solid black coats, although they can also be red (Drakensberger Breeders' Society of South Africa 2023), and the Nguni cattle display various coat colors, including black, brown, and red (Kunene et al. 2022). These coat colours hold cultural and breed-specific significance, particularly within the Nguni ethnic community, influencing selection preferences (Oosthuizen 1996; Kunene et al. 2022). The *TUBB3* gene, identified in the DRB-versus-NGI pair is linked with coat colour regulation (Taye et al. 2018)*,* and melanogenesis which is important for pigmentation and inflammation processes (Taye et al. 2018; Goud et al. 2020). Adaptive mechanisms such as light-colored coats help reflect more solar radiation, reducing heat load (Hansen 2004), and have also been linked to lower tick infestation in the Nguni cattle (Mapholi et al. 2014). The Drakensberger breed has a sleek, shining black coat and is primarily farmed in high altitude regions of South Africa with high solar radiation, where the black coat effectively reflects sunlight and is regarded as an adaptive mechanism in the breed (Rege and Tawah 1999).

Immunity-related genes were also identified in the present study; for example, the *CD14* gene was found in the NGI population and is linked to innate immunity, providing defense against pathogens responsible for mastitis and glomerulonephritis (Lee et al. 2003; Yoon et al. 2003; Pal et al. 2011). In DRB cattle, the *WDPCP* gene is associated with inflammatory responses and disease resistance (de Las Heras-Saldana et al. 2019; Afonso et al. 2020), while the *DTX1* gene, observed in NGI, plays a role in negative regulation of lymphocyte which prevent incidences of autoimmune response (Silva et al. 2022). The *WDPCP* gene has also been linked to inflammatory responses related to claw disorders (interdigital hyperplasia) in Holstein cattle through QTL261577 in the cattle QTL database (Hu et al. 2022; Sölzer et al. 2022). Additionally, the *WDPCP* gene is involved in adipogenesis regulation (Sazzini et al. 2016), and is also linked to GO terms related to high-altitude adaptation (GO:0007224) and respiratory system development (GO:0060541). The *CBFA2T3* gene, upregulated in response to hypoxia (GO:0001666), was identified in DRB-versus-NGI pair and is linked to high-altitude adaptation in Ethiopian indigenous cattle (Terefe et al. 2022). These genes could account for the adaptation of the DRB breed in high altitude areas such as the Drakensberg Mountain (Drakensberger Breeders' Society of South Africa 2023).

The *ELMO3* and *ADAMTS12* genes, identified in DRB, and NGI and TUL, respectively, are linked to phagocytosis, cell migration and in renewal of intestinal epithelia, contributing to immune defense (Nakamura and Mizuno 2010; Cheng et al. 2015). The present study also identified three lysozyme genes, *LYZ1, LYZ2*, and *LYZ3*, in TUL and DRB-versus-NGI comparisons, which are involved in mucosal inflammation and host resistance (Wu et al. 2012; Li et al. 2015). In addition, the *CBFA2T3* and *LY96* genes, associated with inflammation (Alshawi et al*.* 2019), pathogen detection and immune signaling (Dou et al. 2013; Taiwo et al. 2024) were identified in the current study. The selection of these genes implies that the three breeds possess a more effective mechanism for pathogen recognition and immune response.

Several other genes observed were previously associated with immunity in species other than cattle, for example, *SLC4A9*, previously linked to saliva secretion, plays a role in adaptation to dry environments (Peña-Münzenmayer et al. 2015), while *EXOC3L1* and *DEF8* are associated with immune cell function (Siiskonen et al. 2016; Zhang 2023). The *MYO1G* and *MYO1F* genes contribute to phagocytosis and immune cell motility (Maravillas‐Montero et al. 2014; Sun et al. 2021), and *DDX56* has antiviral functions (Taschuk et al. 2020). These genes highlight potential immune and adaptive mechanisms that may warrant further investigation in cattle.

The ROH analysis indicated variations in the number and length of ROH, providing insights into population history and recent admixture. The study highlighted a high prevalence of shorter ROH suggesting selection signatures and ancient inbreeding. The ROHet and F_ST_ analysis identified several genes associated with thermotolerance and disease resistance. Selection signatures identified based on F_ST_, ROH, and ROHet, revealed various genes associated with traits of economic importance.

## Conclusion

Overall, this study enhances the understanding of ROH and ROHet distribution and selection signatures in South African indigenous cattle breeds, providing insights into their genetic mechanisms for drought, disease tolerance, and thermotolerance. The findings highlight the importance of precise inbreeding estimation and provide evidence of possible genetic mechanisms of adaptive traits in SA indigenous breeds. Future studies should include both phenotypic and genotypic data, utilizing higher-density genomic information and longitudinal studies for broader application of the outcomes.

## Supplementary Information

Below is the link to the electronic supplementary material.

Supplementary file1 (DOCX 425 KB)

## Data Availability

The datasets generated and/or analyzed during the current study are not publicly available but are available from the corresponding author on reasonable request.
