# Supplementary material for: Selection signatures associated with adaptation in South African Drakensberger, Nguni, and Tuli beef breeds
Source: Trop Anim Health Prod. 2024 Dec 27;57(1):13. doi: 10.1007/s11250-024-04265-8 (PMC11680604; doi:10.1007/s11250-024-04265-8)
Supplement: Supplementary file 1 — Supplementary file1 (DOCX 425 KB) [file 11250_2024_4265_MOESM1_ESM.docx]

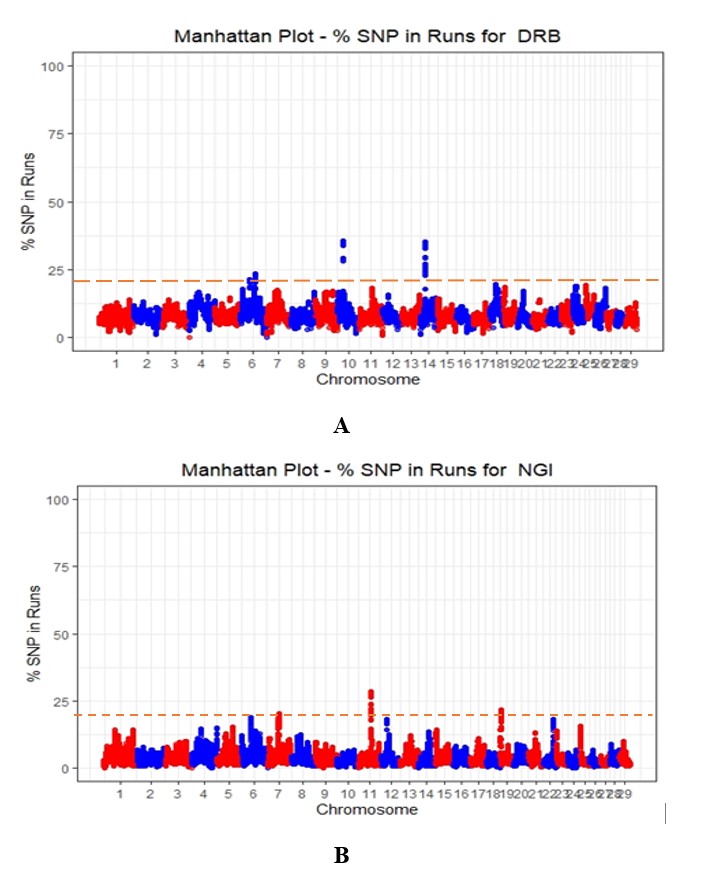


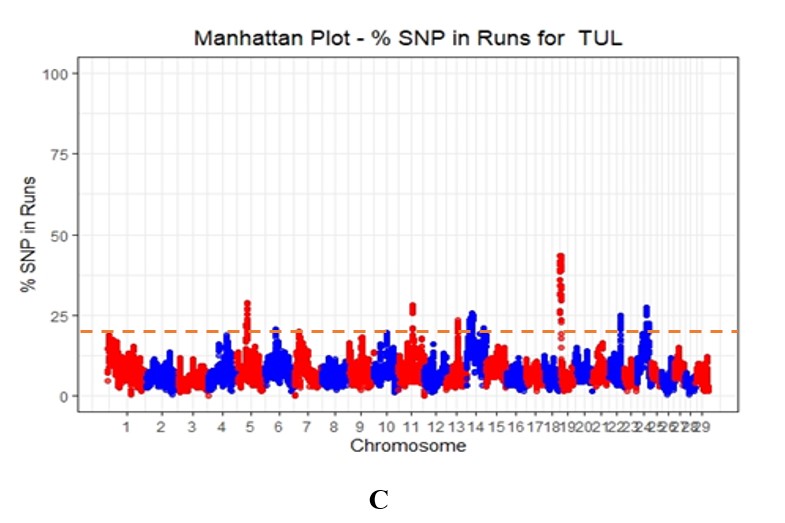


Supplementary **Figure 1A**, **B** and **C**. Manhattan plots for ROH islands distribution across the autosomes in Drakensberger, Nguni, and Tuli Populations. The dotted line represents a threshold of 20% in DRB, NGI, and TUL respectively.


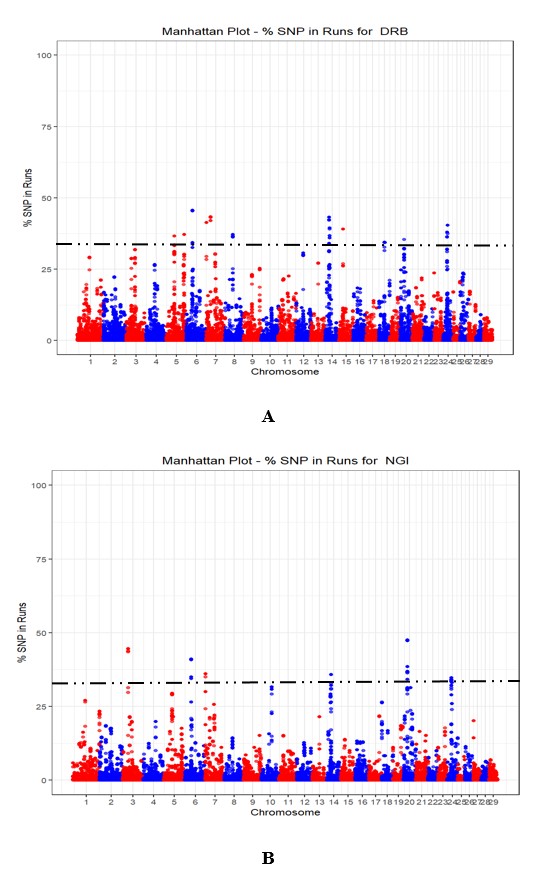


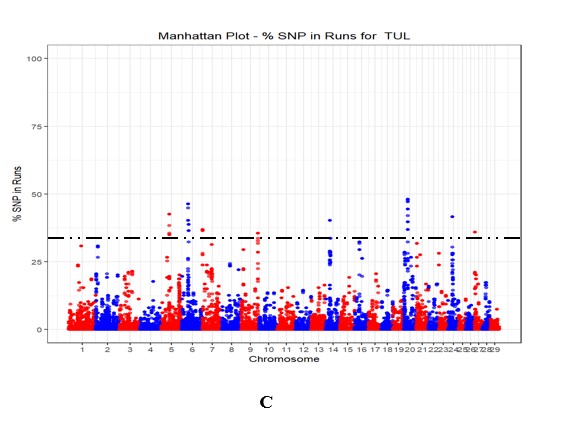


Supplementary **Figure 2 A, B,** and **C** Manhattan plots for ROHet islands distribution across the autosomes in Drakensberger, Nguni, and Tuli Populations. The dotted line represents a threshold of 30% in DRB, NGI, and TUL respectively.


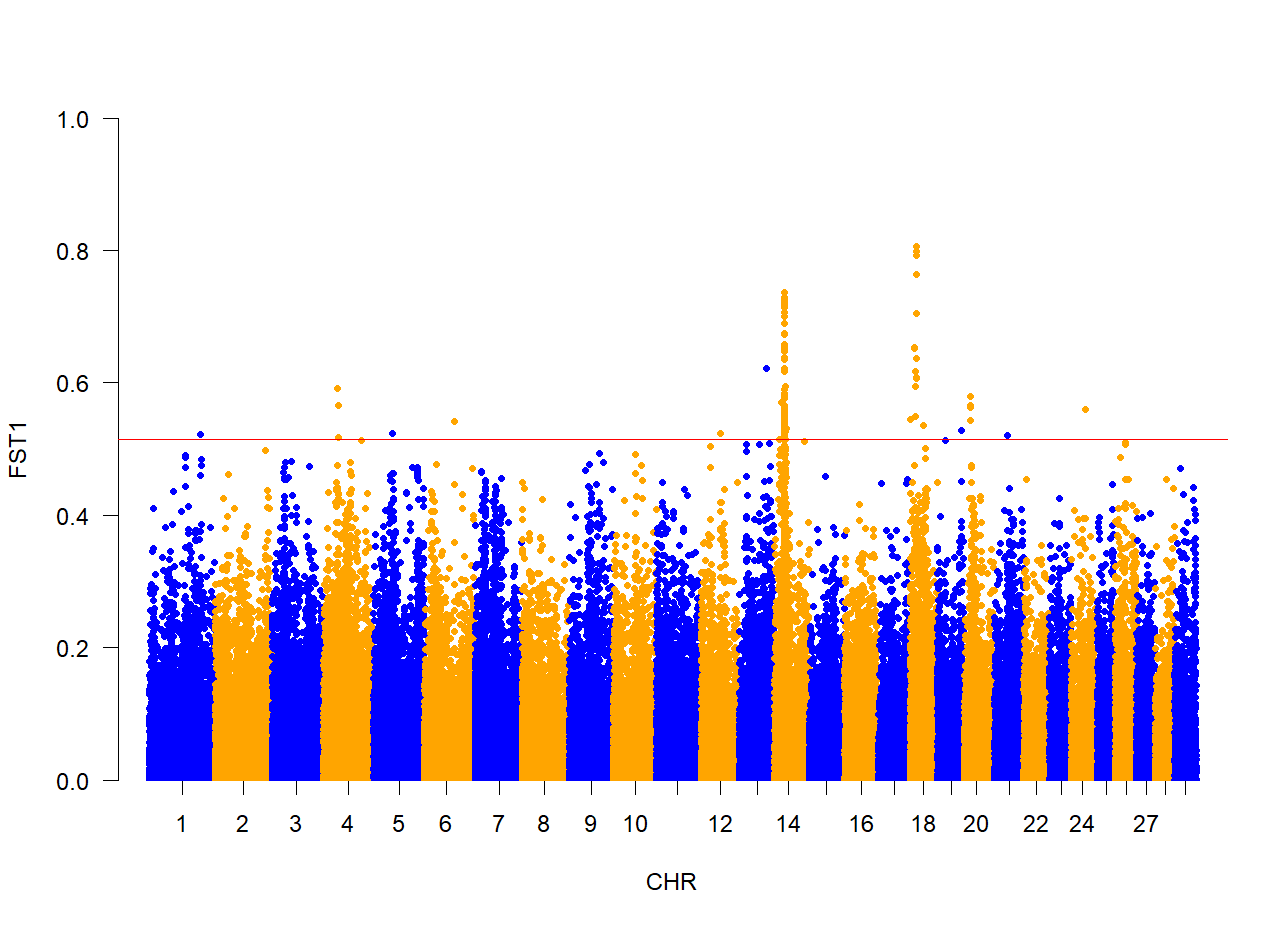


A) DRB vs NGI


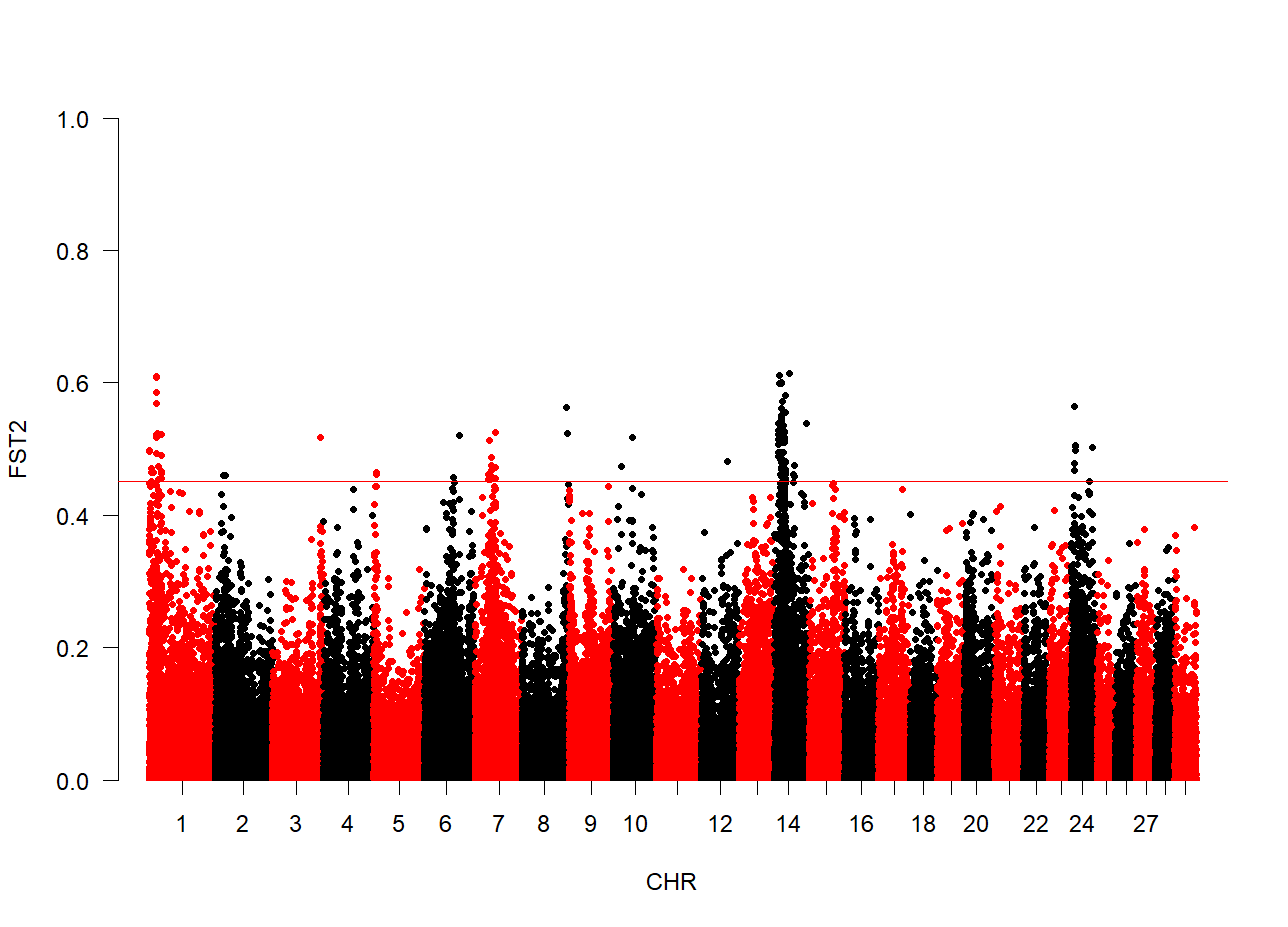


B) NGI vs TUL


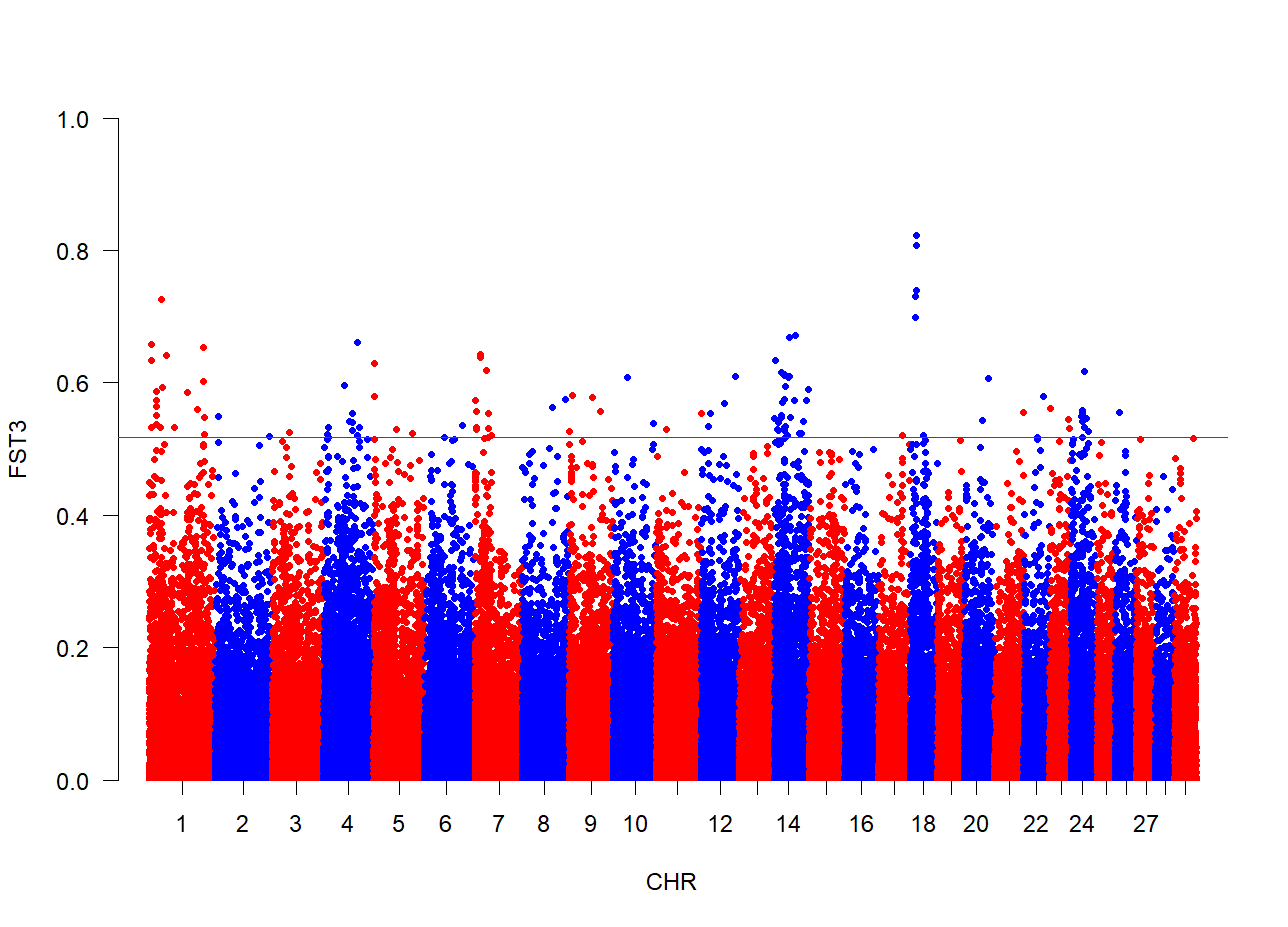


C) DRB vs NGI

Supplementary **Figure 3 A, B** and **C:** Manhattan plots for the distribution of top 0.1 FST values within the autosomes for three breed combinations: A) DRB vs NGI, B) NGI vs TUL, and C) DRB vs TUL
